# Effects of Tibial Nerve Electrostimulation in Patients With Fecal Incontinence: A Systematic Review

**DOI:** 10.1002/pri.70169

**Published:** 2026-02-13

**Authors:** Janaina dos Santos Sóstennes, Tainah Santos Lacerda, Marcos Venicius Bentes do Nascimento, Katiane da Costa Cunha, Rodrigo Santiago Barbosa Rocha, João Simão de Melo Neto, Erica Feio Carneiro

**Affiliations:** ^1^ Universidade do Estado do Pará Belém Pará Brasil; ^2^ Universidade Federal do Pará Belém Pará Brasil

**Keywords:** fecal incontinence, neuromodulation, posterior tibial nerve stimulation, quality of life

## Abstract

**Background and Objective:**

Fecal incontinence (FI) is a condition characterized by the involuntary loss of stool, resulting from the inability to control the sphincter and neuromuscular mechanisms responsible for continence. Percutaneous electrical stimulation of the posterior tibial nerve (posterior tibial nerve stimulation—PTNS) has been used as a therapeutic alternative in the treatment of FI. The objective of this study was to investigate the efficacy.

**Methods:**

A systematic review was conducted according to the recommendations and criteria described in the PRISMA (Preferred Reporting Guide to Systematic Reviews and Meta‐Analyses) items and in the Cochrane Manual. Experimental studies that evaluated the effects of posterior tibial nerve electrostimulation in adult patients with fecal incontinence were included. Case reports, literature reviews, and gray literature were excluded. The search was performed in the MEDLINE/PubMed, Cochrane Library, Scopus, Regional Portal of the VHL, Embase, CINAHL, and Web of Science databases.

**Results:**

Seventeen studies were included, totaling 1248 participants. The average duration of treatment protocols was 12 weeks, with predominantly weekly interventions. The most frequently used stimulation parameters included frequencies between 10 and 20 Hz and pulse widths of 200 μs.

**Discussion:**

Most studies demonstrated a reduction of 50% or more in fecal incontinence episodes, as well as significant improvement in severity scores and quality of life. Randomized clinical trials presented a low risk of bias, while some observational studies demonstrated methodological limitations. PTNS is a minimally invasive intervention with a favorable safety profile and potential clinical applicability.

## 1 Introduction

Fecal continence is a physiological process that depends on the integrity of anatomical structures and the somatic and autonomic nervous systems. The need to evacuate begins with stimuli from the autonomic nervous system through extrinsic nerves, but physiological defecation occurs according to the individual's will, through control of the anal sphincters. From this perspective, it is important to understand that the pelvic floor muscles play a role in maintaining continence, in addition to supporting the pelvic organs and dealing with changes in intra‐abdominal pressure (Queralto et al. 2005).

Fecal incontinence (FI) is a condition in which an individual loses the ability to control bowel movements, resulting in involuntary loss of stool. It can be classified as motor, when the individual feels the urge to defecate but is unable to retain stool; and sensory, when stool loss occurs without the individual noticing. Regardless of the situation, FI can lead to isolation, fear, and insecurity, resulting in physical and psychological damage that directly affects the individual's quality of life (Hotouras et al. 2012).

The causes of fecal incontinence are diverse and may result from disorders of the pelvic floor muscles or nerves, external sphincter dysfunction, surgical or childbirth complications, and neurological or neuropathic disorders. Treatment is multidisciplinary, including medication, diet, psychological support, and physical and functional rehabilitation (Hotouras et al. 2014).

Among the approaches to pelvic physical therapy, neuromodulation or electrical stimulation of the posterior tibial nerve (PTNS) has been widely accepted in the treatment of fecal incontinence, as it is less uncomfortable and causes less embarrassment, since it is not applied in the perianal region. The posterior tibial nerve originates from the sciatic nerve, which originates from the L4, L5, S1, S2, and S3 nerve roots and has sensory, motor, and autonomic fibers (Jiménez‐Toscano et al. 2015). Therefore, when stimulating the posterior tibial nerve, the lumbar and sacral plexuses and the pudendal nerve are also indirectly stimulated by reflex. Electrical stimulation of the tibial nerve should always consider the motor point of the nerve up to the flexion of the hallux or other toes. The parameters vary with an intensity of 10–35 mA, pulse width of 200 µs, and frequency of 10–20 Hz for 30 min (Knowles et al. 2015; Peña Ros et al. 2015; Kelly et al. 2016).

Regarding the placebo effect, Wald (2022) highlight that neuromodulatory interventions, because they involve repeated procedures, frequent therapeutic contact, and high expectations on the part of patients, are particularly susceptible to relevant placebo responses. When analyzing controlled clinical trials with sham stimulation, Wald shows that the improvement observed in the group undergoing active stimulation was not superior to that recorded in the placebo group, suggesting that the clinical benefits reported in previous studies may largely reflect non‐specific effects of the intervention. Thus, the author emphasizes that the absence of sham controls in previous studies likely led to an overestimation of the efficacy of tibial nerve stimulation, reinforcing the need for rigorous methodological designs to distinguish the true therapeutic effect of neuromodulation from the placebo effects inherent in the treatment.

Thus, the hypothesis was raised that the use of posterior tibial nerve electrostimulation may aid in the treatment of dysfunctions related to fecal incontinence, enabling functional improvement and promoting health and quality of life. Therefore, the objective of this systematic review was to investigate the effectiveness of posterior tibial nerve electrostimulation in the treatment of fecal incontinence.

## 2 Materials and Methods

### 2.1 Search Strategy

We conducted a systematic review in accordance with the recommendations and criteria described in the Preferred Reporting Items for Systematic Reviews and Meta‐Analyses (PRISMA) guidelines and the Cochrane Manual. The study protocol was registered in the International Prospective Register of Systematic Reviews (PROSPERO) under number CRD420250653035. The search strategy was developed based on the following guiding question: Is posterior tibial nerve electrostimulation effective in the treatment of fecal incontinence?

From there, we used the PICO strategy: P: fecal incontinence; I: posterior tibial nerve stimulation; C: not specified; O: quality of life/quality of life indicators. The strategy was constructed using descriptors contained in the Health Sciences Descriptors (DeCS/MeSH) and Health Sciences Descriptors (DEC). The search strategy was adapted to be applied to each of the databases used in the research.

### 2.2 Eligibility Criteria

Experimental studies evaluating the effects of electrostimulation on the posterior tibial nerve in adult patients with fecal incontinence were included; there were no language/linguistic or time period restrictions. Case reports, literature reviews, and gray literature were excluded.

### 2.3 Data Sources

The included studies were identified through a high‐sensitivity search conducted in the following databases: MEDLINE/PubMed, Cochrane Library, Scopus, Regional VHL Portal, Embase, CINAHL, and Web of Science. The search strategy involved cross‐referencing selected keywords in English and Spanish. The complete search strategy, including all descriptors, Boolean operators, and filters applied, is presented in Appendix A as Table S1.

### 2.4 Data Extraction

After searching the databases, the files of the articles found were included in Rayyan QCRI. In this study screening resource, the articles were selected according to eligibility criteria by three researchers (JSS, TLS, and MVBN) in a blinded manner, with conflicts being resolved by a third researcher (RSBR). The selected articles were read in full. The data were extracted and tabulated in Microsoft Excel.

### 2.5 Study Quality Assessment

The ROB 2 tool was used to assess the quality of studies and the risk of bias for randomized clinical trials, and the Newcastle‐Ottawa scale was used for other studies. The assessment was performed by three researchers (JSS, TLS, and MVBN) in a blinded manner; disagreements were resolved by consensus among them.

## 3 Results

**FIGURE 1:**
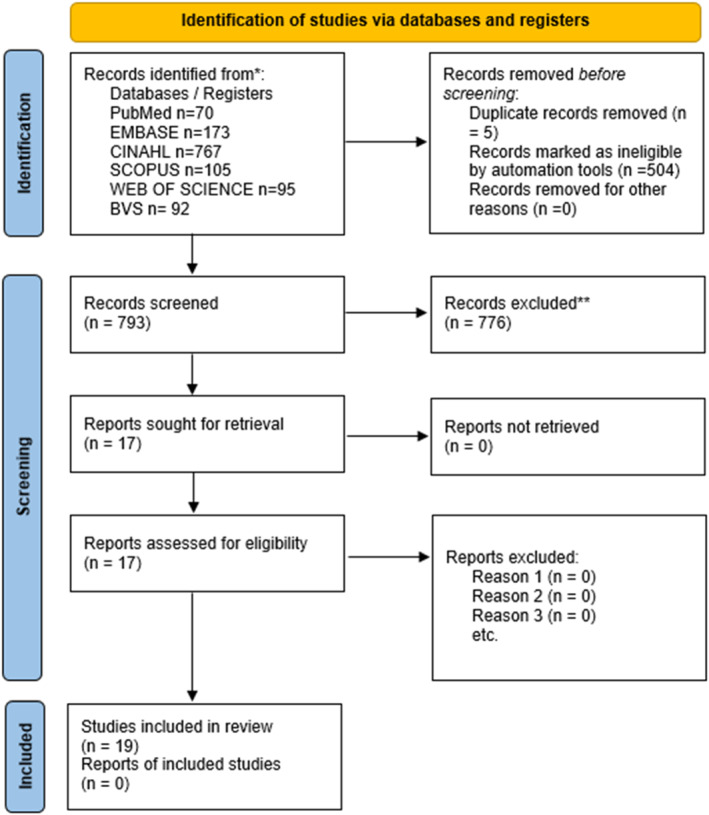
PRISMA flowchart.

After selecting studies, removing duplicates, and applying eligibility criteria, as described in the PRISMA flowchart (Figure 1), 17 studies were included in this systematic review, totaling 1248 participants with fecal incontinence refractory to conservative treatment. The studies showed significant clinical improvement in different aspects related to the severity of fecal incontinence (FI), frequency of episodes, and quality of life.

Table 1 presents the main characteristics of the included studies. Most of the research was conducted in Europe, especially in the United Kingdom, Spain, and France, followed by studies conducted in Australia and Japan, totaling 1248 participants undergoing posterior tibial nerve stimulation (PTNS). The sample size ranged from 10 to 227 participants, and the studies were published between 2005 and 2024. Only two studies had a randomized clinical trial design.

**TABLE 1:** Characterization of the studies included in the review.

Author (year)	Country where the study was conducted	Population	Outcome	Interventions
Queralto et al. (2005)	France	10	60% improvement in FI score.	PTNS
Hotouras et al. (2012)	United Kingdom	100	Reduction of up to 80%–100% in episodes of fecal incontinence and statistically significant improvement in severity scores (CCF‐FI) and quality of life (FIQL) in patients with urge and mixed incontinence, while in passive incontinence there was no significant improvement.	PTNS
Jiménez‐Toscano et al. (2015)	Spain	27	A 29% decrease in emergency episodes; IF changed to solid stools in 48.1% of patients and a statistically significant improvement in lifestyle.	PTNS
Knowles et al. (2015)	United Kingdom	227	50% or greater reduction in the average number of episodes of FI.	PTNS
Peña Ros et al. (2015)	Spain	55	Improvement of at least 50% in the Wexner score.	PTNS
Kelly et al. (2016)	United Kingdom	79	78.5% reported symptomatic improvement.	PTNS
Dedemadi and Takano (2018)	Japan	22	Redução de 50% nos episódios de insuficiência intestinal, diminuição na pontuação de Wexner e melhora nas pontuações do FIQL.	PTNS
Hidalgo‐Pujol et al. (2018)	Spain	56	50% reduction in baseline CCIS.	PTNS
Sanagapalli et al. (2018)	United Kingdom	33	Significant reduction in Wexner score (from 13.5 to 7.0), improvement in quality of life, and improvement in stool consistency. RRMS had the highest success rate (95%).	PTNS
Manso et al. (2020)	Spain	73	39% of patients showed improvement in retention time and a significant increase in manometric results—maximum resting and compression pressure.	PTNS
Mazor et al. (2020)	Austrália	29	50% reduction in episodes of FI per week and improvement in FISI and VAS scores.	PTNS + BF
Álamo Vera et al. (2020)	Spain	21	33.3% reduction in episodes of HF and statistically significant improvement in Wexner and FIQL.	PTNS
Leo et al. (2021)	United Kingdom	50	50% or greater reduction in the average number of episodes of FI per week.	PTNS
Bosch‐Ramírez et al. (2023)	Spain	139	Reduction in fecal urgency, increasing to 89.8% the number of patients able to delay bowel movements for more than 10 min, decreased the Wexner score from 11 to 4 in 36 months, and significantly improved the severity of incontinence and the “embarrassment” domain of quality of life.	PTNS
Luchristt et al. (2023)	United States	166	≥ 50% Reduction in fecal incontinence episodes (FIEs) recorded in the bowel diary (14 days).	PTNS
O'Connor, Reynolds, et al. (2024)	United Kingdom	61	≥ 50% Improvement in scores or episodes.	PTNS
O’Connor, Molyneux, et al. (2024)	United Kingdom	135	≥ 50% Improvement in symptoms (defined as “success”); change in SMIS and MHQ scores; variation in manometric parameters.	PTNS

Abbreviations: FI, fecal incontinence; PTNS, transcutaneous posterior tibial nerve stimulation.

The characteristics of the study population are presented in Table 2. The mean age of participants ranged from 48 to 67 years, with a predominance of females in most studies, and there was also a study with a population composed exclusively of women (Mazor et al. 2020; Luchristt et al. 2023). Most studies included individuals who had previously failed conservative treatment, including interventions such as behavioral guidance, medication, and biofeedback. Some studies evaluated specific populations, such as patients with multiple sclerosis, and the presence of refractory FI was the most prevalent criterion among the studies.

**TABLE 2:** Characterization of the population.

Author (year)	Population	Eligibility criteria	Number of women	Average age
Queralto et al. (2005)	10	Absence of ultrasonographic sphincter defect; absence of anatomical rectal prolapse; clinical failure of medical treatments and biofeedback rehabilitation.	10	61.7
Hotouras et al. (2012)	100	Adult patients with fecal incontinence (urge, passive, or mixed) refractory to conservative treatment, previously evaluated with clinical examination, colonoscopy, and anorectal tests, and able to participate in 12 PTNS sessions and complete clinical records.	88	57
Jiménez‐Toscano et al. (2015)	27	Failure of conservative treatment, episodes of FI for more than 6 months, Werner score of more than 5, and at least 5 episodes of FI in the 21‐day fecal diary.	31	67
Knowles et al. (2015)	227	IF severe enough to warrant intervention; previous conservative therapies ineffective.	205	58.0
Peña Ros et al. (2015)	55	Adults over 18 years of age; FI greater than 6 months; failure of conservative treatment; adequate motor and sensory response; able to understand the investigator's orders; FI causing disruption to quality of life.	44	58.6
Kelly et al. (2016)	79	Diagnosis of IF; failure to respond to biofeedback; patient able to attend 12 appointments; accepts that additional refresher treatments may be necessary after the initial 12‐week course of treatment; referral form completed by colorectal consultant.	79	57.5
Dedemadi and Takano (2018)	22	Patients with at least 6 months of FI.	13	64
Hidalgo‐Pujol et al. (2018)	56	Age over 18, failure of previous treatment.	38	59
Sanagapalli et al. (2018)	33	Patients with multiple sclerosis and refractory fecal incontinence, who had already failed conservative therapies and undergone anorectal evaluation.	25	48
Manso et al. (2020)	73	Adult patient with any degree of FI.	57	60
Mazor et al. (2020)	29	Women, aged between 18 and 80, with FI 2 to 4 per month in the last 6 months, minimum score of 8 on the FISI score.	13	54
Álomo et al. (2020)	21	Patients over 18 years of age who do not respond to conventional treatment.	11	51
Leo et al. (2021)	50	Adults over 18 years of age; passive or mixed FI; minimum of 2 or more episodes of FI per week; failure of other treatments; able to self‐administer the renew anal insert.	24	56.0
Bosch‐Ramírez et al. (2023)	139	Age ≥ 18 years, diagnosis of fecal incontinence (FI) defined as: ≥ 1 episode of fecal incontinence per week for more than 6 months, FI refractory to conservative treatment.	110	63
Luchristt et al. (2023)	166	Women ≥ 18 years of age with at least 3 months of ABL (accidental bowel leakage) symptoms and a St. Mark score ≥ 12.	166	64
O'Connor, Reynolds, et al. (2024)	61	Patients with IF who received both treatments and had complete data.	12	61
O’Connor, Molyneux, et al. (2024)	135	Patients with FI; clinical and manometric data before and after PTNS available.	119	60

Abbreviation: FI, fecal incontinence.

**FIGURE 2:**
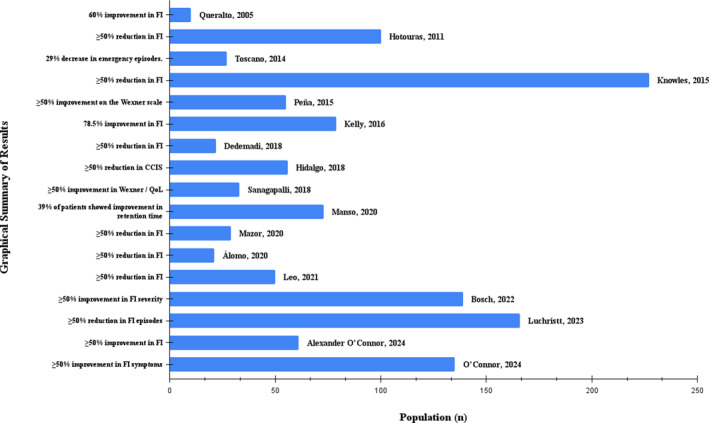
Graphical summary of the main quantitative results.

Table 3 describes the characteristics of the interventions. The duration of treatment ranged from 4 weeks to 12 months, with 12 weeks being the most frequently used protocol. Regarding electrostimulation parameters, the most commonly used frequency was 10–20 Hz, with a pulse width of 200 μs in all studies. The application was predominantly performed once a week, lasting 30 min. Figure 2 presents a graphical summary of the main clinical outcomes observed. Regarding clinical outcomes, all 17 included studies reported improvement in fecal incontinence symptoms after posterior tibial nerve stimulation intervention. The most frequently evaluated outcomes were a reduction in the number of episodes of fecal incontinence, a decrease in severity scores, including Wexner, Cleveland Clinic Incontinence Score (CCIS), Fecal Incontinence Severity Index (FISI), and improvement in quality of life, measured predominantly by the Fecal Incontinence Quality of Life Questionnaire (FIQL). A reduction in the number of weekly episodes of incontinence was observed in studies with different designs and sample sizes, with several studies adopting a reduction of 50% or more as the criterion for clinical response, an outcome achieved in a significant proportion of participants. Significant reductions in severity scores were also reported, with clinically relevant absolute or percentage decreases in the instruments used. At the same time, studies that assessed quality of life demonstrated consistent improvement in physical, emotional, and social domains after treatment, particularly in domains related to symptom severity, behavior, and social embarrassment.

**TABLE 3:** Characterization of the intervention.

Author (year)	Study design	Electrode type	Total duration	Frequency (Hz)	Pulse width (μs)	Sessions (*n*/frequency)
Queralto et al. (2005)	Case series	Surface	4 weeks	10	200	Diary; 20 min
Hotouras et al. (2012)	Prospective cohort	Surface	12 weeks	20	200	Weekly; 30 min
Jiménez‐Toscano et al. (2015)	Prospective study	Surface	12 weeks	10	200	Weekly; 30 min
Knowles et al. (2015)	RCT	Needle	13 weeks	10	200	Weekly; 30 min
Peña Ros et al. (2015)	Prospective study	Needle	12 weeks	20	200	Weekly; 30 min
Kelly et al. (2016)	Cohort	Needle	12 weeks	20	200	Weekly; 30 min
Dedemadi and Takano (2018)	Prospective cohort	Surface	6 weeks	10	200	Weekly; 30 min
Hidalgo‐Pujol et al. (2018)	Prospective cohort	Needle	12 weeks	20	200	Weekly; 30 min
Sanagapalli et al. (2018)	Prospective study	Needle	8 weeks	NR	NR	Weekly; 30 min
Manso et al. (2020)	Prospective study	Surface	12 weeks	20	NR	Weekly; 30 min
Mazor et al. (2020)	Prospective study	Needle	13 weeks	NR	NR	Weekly; 30 min biofeedback + PTNS
Álamo Vera et al. (2020)	Prospective study	Surface + needle	12 weeks	20	200	Weekly; 30 min
Leo et al. (2021)	Pilot study	Needle	12 weeks	10	200	Weekly; 30 min
Bosch‐Ramírez et al. (2023)	Prospective study	Needle	12 weeks	20	200	Weekly; 30 min
Luchristt et al. (2023)	Multicenter RCT	Needle versus sham	12 weeks	NR	NR	Weekly; 30 min
O'Connor, Reynolds, et al. (2024)	Retrospective	Surface + needle	12 weeks	NR	NR	Weekly; 30 min
O’Connor, Molyneux, et al. (2024)	Retrospective	Surface	12 weeks	NR	NR	Weekly; 30 min

Abbreviation: NR, unreported.

**FIGURE 3:**
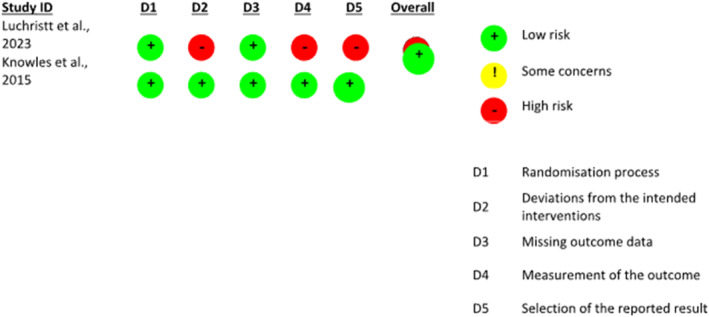
Assessment of risk of bias in the included randomized clinical trials, according to the ROB 2 tool (Cochrane).

According to the ROB 2 tool (Figure 3), the study by Luchristt et al. (2023) presented a low risk of bias in all domains evaluated. The CONFIDeNT trial (Knowles et al. 2015) was classified as presenting some concerns, mainly related to the selection of reported results, which reinforces the need for cautious interpretation of the findings, especially in the context of interventions susceptible to the placebo effect. In the Newcastle‐Ottawa Scale (NOS) assessment, presented in Table 4, non‐randomized studies scored between 3 and eight points, indicating heterogeneity in methodological quality. The study by Bosch‐Ramírez et al. (2023) had the highest score (6/9). In contrast, other studies show lower ratings, suggesting a higher risk of bias.

**TABLE 4:** Methodological quality assessment (Newcastle‐Ottawa scale).

Author (year)	Is the case definition adequate?	Representativeness of the cases	Selection of controls	Definition of controls	Comparability of cases and controls on the basis of the design or analysis	Ascertainment of exposure	Same method of ascertainment for cases and controls	Non‐response rate	Total
Queralto et al. (2005)	*	*		*		*			4/9
Hotouras et al. (2012)	*				*	*	*	*	5/9
Jiménez‐Toscano et al. (2015)	*	*		*	*	*			5/9
Peña Ros et al. (2015)	*	*				*			3/9
Kelly et al. (2016)	*						*	*	3/9
Dedemadi and Takano (2018)	*	*			*	*			4/9
Hidalgo‐Pujol et al. (2018)	*	*		*	*	*			5/9
Sanagapalli et al. (2018)	*				*	*	*	*	5/9
Manso et al. (2020)	*	*		*	*	*			5/9
Mazor et al. (2020)	*	*		*	*	*			5/9
Álamo Vera et al. (2020)	*			*	*	*			4/9
Leo et al. (2021)	*	*		*	*	*			5/9
Bosch‐Ramírez et al. (2023)	*	*			*	*	*	*	6/9
O'Connor, Reynolds, et al. (2024)	*	*			*		*	*	5/9
O’Connor, Molyneux, et al. (2024)	*	*			*	*			4/9

## 4 Discussion

The results of this systematic review show that posterior tibial nerve stimulation (PTNS) has positive therapeutic effects in reducing fecal incontinence (FI) in adult populations, especially those refractory to conservative treatment. Overall, the included studies demonstrated clinical improvement in different outcomes, including reduced frequency of FI episodes, decreased severity scores (Wexner, CCIS, FISI), and improved quality of life as assessed by validated instruments (FIQL, MQHQ). Even in the face of the methodological heterogeneity observed, the convergence of results over time reinforces the therapeutic potential of PTNS.

Consistently, the literature analyzed report response rates above 50% in most studies, regardless of the technique used, whether by surface electrodes or percutaneously. Studies such as those by Knowles et al. (2015), Peña Ros et al. (2015), Leo et al. (2021), O'Connor, Reynolds, et al. (2024), and O’Connor, Molyneux, et al. (2024) demonstrated reductions of ≥ 50% in episodes of fecal incontinence, including in populations with long‐term or refractory FI. These findings are particularly relevant considering that PTNS is a minimally invasive, low‐risk intervention that is potentially applicable on a large scale, including in outpatient settings.

The improvement in incontinence severity scores was also consistent across studies. Significant reductions were observed in studies such as Sanagapalli et al. (2018), Bosch‐Ramírez et al. (2023), and Mazor et al. (2020), suggesting that PTNS may act on both sensory‐motor and behavioral components related to sphincter control. In addition, studies that incorporated objective physiological assessments, such as anorectal manometry, showed increased resting and voluntary contraction pressures, indicating a possible distal neuromuscular modulation mechanism associated with the therapy.

Another relevant aspect refers to the improvement in quality of life, documented in studies that used specific instruments such as the FIQL. Research conducted by Hotouras et al. (2012), Jiménez‐Toscano et al. (2015), and Dedemadi and Takano (2018) demonstrated significant improvement in domains related to lifestyle, social embarrassment, and emotional impact, reinforcing that the benefits of PTNS go beyond objective clinical outcomes and reach psychosocial and functional dimensions that are important to the patient.

With regard to stimulation parameters, there is relative convergence among studies, with most adopting frequencies between 10 and 20 Hz, pulse width of 200 µs, weekly sessions of 30 min, and a duration of approximately 12 weeks. This protocol appears to be associated with higher clinical response rates. In multimodal approaches, such as in the study by Mazor et al. (2020), in which PTNS was associated with anorectal biofeedback, additional benefits were observed, suggesting a possible synergistic effect.

Despite these favorable findings, the methodological quality of the studies varied considerably. While some randomized clinical trials demonstrated low risk of bias, most observational studies had frequent limitations, such as lack of a control group, risk of selection bias, and difficulty in controlling for confounding factors. Nevertheless, the consistency of results over nearly 2 decades lends clinical relevance to the available body of evidence.

Heterogeneity between studies was assessed qualitatively and quantitatively. Although the *I* ^2^ index is commonly used to quantify statistical heterogeneity in meta‐analyses, its application was not possible in this review, since the included studies presented high clinical and methodological heterogeneity, in addition to incomparable outcomes and metrics. The studies differed substantially in terms of design, population, definition of therapeutic success, measurement instruments, and stimulation parameters, making it impossible to extract estimates of combination effects. Thus, performing a meta‐analysis and calculating the *I* ^2^ would not be methodologically appropriate, and a narrative synthesis of the results was chosen, as recommended by the PRISMA guidelines and the Cochrane Handbook.

However, the interpretation of the specific efficacy of posterior tibial nerve stimulation (PTNS) should be made in light of evidence from randomized placebo‐controlled clinical trials. As discussed by Wald (2022), neuromodulation interventions are particularly susceptible to placebo effects due to frequent therapeutic interaction, patient expectations, and the procedural nature of the treatment itself. The randomized clinical trial conducted by Horrocks et al. (2014) demonstrated that PTNS was not superior to sham stimulation after 12 weeks of treatment, despite the clinical improvement observed in both groups. Convergently, the multicenter study conducted by Luchristt et al. (2023) reinforced this finding by showing no statistically significant difference between active PTNS and sham in the primary outcomes, especially when objective measures of therapeutic success were evaluated. Together, these results indicate that a substantial portion of the clinical response observed can be attributed to non‐specific effects of the intervention, suggesting that uncontrolled studies tend to overestimate the actual efficacy of PTNS.

In this context, the variability of protocols observed in the literature can be explained by the still exploratory nature of tibial neuromodulation in the treatment of fecal incontinence and by the historical absence of consensus based on robust evidence. Differences in methodological design, electrode type, treatment duration, session frequency, and electrical parameters reflect empirical attempts to adapt to different clinical contexts and pathophysiological profiles, which makes it difficult to define reliable dose‐response relationships.

Furthermore, it is not possible to state that stimulation by surface electrodes is superior to percutaneous needling or vice versa. The absence of clinical trials specifically designed to compare these modalities, combined with the heterogeneity of outcomes and the influence of the placebo effect demonstrated in controlled studies, prevents definitive conclusions regarding technical superiority.

Another relevant limiting factor is the lack of standardized assessment of sphincter integrity. Many studies do not systematically describe the anatomical and functional status of the anal sphincters, nor do they use uniform methods such as endoanal ultrasound or anorectal manometry with well‐defined criteria, which compromises the adequate stratification of participants and hinders the identification of subgroups that are potentially more responsive to neuromodulation.

Finally, the findings of this review are consistent with previous systematic reviews, such as those by Edenfield et al. (2015) and Horrocks et al. (2014), which already suggest clinical improvement associated with tibial nerve stimulation, but highlight relevant methodological limitations and the scarcity of randomized controlled clinical trials. Complementarily, the meta‐analysis conducted by Sarveazad et al. (2019), when comparing percutaneous tibial nerve stimulation with simulated stimulation, identified a significant reduction in the number of episodes of fecal incontinence, but without consistent differences in severity scores or manometric parameters, reinforcing the variability of the outcomes evaluated and the uncertainty regarding the magnitude of the therapeutic effect. In addition, the systematic review with meta‐analysis by Simillis et al. (2018), which compared posterior tibial nerve stimulation with sacral nerve stimulation, demonstrated the superiority of sacral stimulation in functional outcomes and specific domains of quality of life, while also highlighting clinical heterogeneity, inconsistency in PTNS protocols, and risk of bias in the included studies. In summary, PTNS is a safe and feasible intervention with potential benefits; However, the consolidation of definitive clinical recommendations remains limited by the quality and heterogeneity of the available evidence, requiring multicenter randomized clinical trials, strictly controlled by sham, with adequately sized samples, prolonged follow‐up, and standardization of stimulation parameters and clinical outcomes.

Furthermore, compared to previously published systematic reviews, the present study advances by integrating more recent evidence, including multicenter randomized placebo‐controlled clinical trials and contemporary observational studies, allowing for a more comprehensive and up‐to‐date analysis of posterior tibial nerve stimulation in the treatment of fecal incontinence. Unlike previous reviews, which were predominantly based on case series or a limited number of randomized trials, this review critically emphasizes the role of the placebo effect in the observed clinical response. In addition, the present study contributes methodologically by demonstrating that the high clinical, methodological, and outcome heterogeneity—associated with the variability of stimulation protocols, the lack of standardization in the assessment of sphincter integrity, and the diversity of therapeutic success criteria—makes it impossible to perform a robust meta‐analysis. Thus, rather than reaffirming the potential benefits of PTNS, this study qualifies the available evidence, more precisely defining its limitations and highlighting critical gaps that should guide the design of future randomized clinical trials, especially in the context of physical therapy practice.

## 5 Implications of Physical Therapy Practice

Posterior tibial nerve stimulation (PTNS) may be associated with improvement in fecal incontinence symptoms in selected patients, especially those who have not responded satisfactorily to conservative therapies. Overall, the included studies suggest a reduction in the frequency of fecal loss episodes, improved severity scores—such as the Wexner scale—and a favorable impact on quality of life, in addition to an apparently adequate safety profile. However, these findings should be interpreted with caution as the available evidence presents heterogeneous and methodologically inconsistent results.

The high variability between studies, involving differences in methodological design, stimulation parameters, frequency and duration of protocols, inclusion and exclusion criteria, as well as the lack of standardization in the assessment of anal sphincter integrity and manometric parameters, limits the comparability of results and made it impossible to perform a robust meta‐analysis.

Thus, although PTNS represents a potentially promising therapeutic strategy, current evidence is still insufficient to support definitive clinical recommendations. It is essential to conduct multicenter, placebo‐controlled randomized clinical trials with standardized stimulation protocols and uniform clinical outcomes to clarify the true magnitude of the therapeutic effect of PTNS, identify subgroups of patients with a higher probability of response, and more accurately define its role in the management of fecal incontinence in the short, medium, and long term.

## Author Contributions

All authors contributed substantially to this manuscript.

## Funding

The authors have nothing to report.

## Ethics Statement

The authors have nothing to report.

## Consent

The authors have nothing to report.

## Conflicts of Interest

The authors declare no conflicts of interest.

## Supporting information


**Table S1:** Search strategy.

## Data Availability

All data are fully available to the authors without restriction.
