# Supplementary material for: Effects of Tibial Nerve Electrostimulation in Patients With Fecal Incontinence: A Systematic Review
Source: Physiother Res Int. 2026 Feb 13;31(2):e70169. doi: 10.1002/pri.70169 (PMC12902902; doi:10.1002/pri.70169)
Supplement: Supplementary file 1 — Table S1: Search strategy. [file PRI-31-e70169-s001.docx]

**Table suplementay 1.** Search strategy

The complete search strategies, with descriptors, Boolean operators, and combinations used in each database, are presented below, including the MeSH and DeCS terms employed. This detail allows for the reproducibility of the review and ensures methodological transparency.

| **Database** | **Descriptors/Search strategy** |
| --- | --- |
| PubMed/MEDLINE | ("Fecal Incontinence"[Mesh] OR "fecal incontinence"[tiab] OR "faecal incontinence"[tiab] OR "incontinência fecal"[tiab]) AND ("Tibial Nerve Stimulation"[Mesh] OR "posterior tibial nerve stimulation"[tiab] OR "percutaneous tibial nerve stimulation"[tiab] OR "transcutaneous tibial nerve stimulation"[tiab] OR "PTNS"[tiab] OR neuromodulation[tiab]) AND ("Quality of Life"[Mesh] OR "quality of life"[tiab] OR "health-related quality of life"[tiab] OR "meaning in life"[tiab] OR "indicators of quality of life"[tiab] OR "qualidade de vida"[tiab]) |
| Embase | ('fecal incontinence'/exp OR 'fecal incontinence' OR 'faecal incontinence' OR 'incontinência fecal') AND ('tibial nerve stimulation'/exp OR 'posterior tibial nerve stimulation' OR 'percutaneous tibial nerve stimulation' OR 'transcutaneous tibial nerve stimulation' OR PTNS OR neuromodulation) AND ('quality of life'/exp OR 'quality of life' OR QoL OR 'qualidade de vida') |
| Cinahl | (MH "Fecal Incontinence+") OR "fecal incontinence" OR "faecal incontinence" OR "incontinência fecal" AND (MH "posterior tibial nerve stimulation" OR "percutaneous tibial nerve stimulation" OR "transcutaneous tibial nerve stimulation" OR PTNS OR neuromodulation) AND (MH "Quality of Life+" OR "health-related quality of life" OR "meaning in life" OR QoL OR "indicators of quality of life" OR "qualidade de vida") |
| Scopus | (TITLE-ABS-KEY("fecal incontinence" OR "faecal incontinence" OR "incontinência fecal")) AND (TITLE-ABS-KEY("posterior tibial nerve stimulation" OR "percutaneous tibial nerve stimulation" OR "transcutaneous tibial nerve stimulation" OR PTNS OR neuromodulation)) AND (TITLE-ABS-KEY("quality of life" OR "health-related quality of life" OR "meaning in life" OR QoL OR "indicators of quality of life" OR "qualidade de vida")) |
| Web of Science | TS=("fecal incontinence" OR "faecal incontinence" OR "incontinência fecal") AND TS=("posterior tibial nerve stimulation" OR "percutaneous tibial nerve stimulation" OR "transcutaneous tibial nerve stimulation" OR PTNS OR neuromodulation) AND TS=("quality of life" OR "health-related quality of life" OR "meaning in life" OR QoL OR "indicators of quality of life" OR "qualidade de vida") |
| BVS / Portal REgional | ("fecal incontinence" OR "faecal incontinence" OR "incontinência fecal") AND ("posterior tibial nerve stimulation" OR "percutaneous tibial nerve stimulation" OR "estimulação do nervo tibial posterior" OR PTNS OR neuromodulation) AND ("quality of life" OR "health-related quality of life" OR "meaning in life" OR "qualidade de vida" OR "indicators of quality of life" OR "qualidade de vida" OR QoL) |

**Note:** The strategy was adapted according to the indexing language and filters available in each database. There were no language restrictions or time limits.
